# Research: a priority for the decade of healthy aging

**DOI:** 10.25100/cm.v50i2.4613

**Published:** 2019-06-30

**Authors:** Enrique Vega Garcia

**Affiliations:** Pan American Health Organization/World Health Organization (PAHO/WHO), Head of the Unit, Healthy Life Course, Washington, DC, USA.

**Keywords:** Healthy aging, elderly, Colombia, life expectancy, longevity, population dynamics, aged, disabled persons, demography, aging, Envejecimiento saludable, adulto mayor, Colombia, esperanza de vida, longevidad, dinámica de población, vejez, discapacidad, demografía, envejecimiento

It is a great opportunity to be part of the publication of this special issue dedicated to aging and health; I thank the editors for this opportunity. The relevance of this publication is not accidental, population aging is undoubtedly one of the most formidable challenges that public health must face in this century. It is a global challenge, but for the Americas, and especially for Latin America and the Caribbean, it is already an inescapable one.

The last decade has been important for public health and aging in the Americas. With pride, we can affirm that in these ten years, no Region has advanced as much as ours. In 2009, the Ministers of Health of the Americas were pioneers in approving an action plan on Aging and Health, the first in the world; in addition, the Americas was the first, and so far the only region in the world, to approve a protection mechanism for the rights of the elderly, the Inter-American Convention on the Rights of Older Persons, in 2015.

Many countries in the Region have reported significant results and advances in the work on the health of the elderly; and have played an important role in the approval of the Global Plan for Aging and Health in 2016 at the World Health Assembly. Today, they take the lead in promoting the Declaration of the Decade of Healthy Aging. However, the depth and speed of the demographic transition in the Americas has overwhelmed our efforts to date. If in 2017 the population in our continent had 14.6% of people aged 60 years and more, by 2050 this figure will reach 25%. The life expectancy of the Region is expected to continue to increase for all ages. The life expectancy at birth today is 77.07 years, the hope of "geriatric life", which begins when people reach 60 years of age, is to live 22.38 years; and in the Americas, a person who reaches 80 years today, will live an average of 9.41 years more. But, perhaps the most important thing is that this evolution will take 35 years for Latin America and the Caribbean; almost half of the time that it took for other regions: it took 65 years for Europe, and 75 years for the United States and Canada ^1^.

Although reducing mortality and prolonging life should be recognized as one of the great results of social development and public health, not everything has been good news. In 2015, it was estimated that the “healthy life expectancy” in the Region was 66.45 years, with a difference of more than 10 years regarding life expectancy (77.07 years). In the Americas, the years lived with disabilities has increased in 12.6%, since 2009. Although there are notable differences among countries, the gap between the two indicators has increased for all of them. Several studies support that more than eight million people aged 60 years or older are dependent, which represents more than 1% of the total population of the region, and 12% of individuals aged over 60 years. It is estimated that by 2050, this figure will triple, so it will be between 27 and 30 million ^2^. 

In spite of the important advances in the Region, in the last decade, the rapidity of the demographic transition, with many other priorities to attend and in a complex socio-economic context, will require a much faster adaptation of health systems. The so-called “window of demographic opportunity” is rapidly narrowing for the Americas; and although the sense of priority on the subject has grown, a greater effort should be made in order to address this transition. The above will require actions and interventions from PAHO/WHO, Member States, the Secretariat and other strategic partners, which would that allow longevity and aging can be a positive result of sustainable development in the Americas ^3^. The above implies that efforts should be redoubled to bridge the gap; there must be learned about the new needs, in order to impact on the conditions that cause large periods of time living with disability and dependence.

In more than 30 years of my career associated with this issue, I have witnessed the effort in Latin America and the Caribbean to create an increasingly solid scientific ground that will contribute to informed and evidence-based decision making by our managers in health; and to evaluate that these interventions really generate important improvements in the health and well-being of the aging population. But it has failed to recognize with greater strength the relevance and influence of these efforts, as the basis of our advances in public policy on aging. This publication is a great space to recognize the pioneering efforts with the SABE study (Health, Welfare and Aging) under the leadership of Dr. Martha Peláez, made by PAHO and other strategic partners at the beginning of this century ^4^. This multinational study was for many years one of the most important sources of the scientific base, which with data and evidence supported the need for the advancement of work in the health of the elderly at all levels and sectors of society. Its influence was extended and stimulated that national studies of transcendental importance were performed, of which the results of this Colombia Medica special issue publication, how a valuable example.

The work is still perfectible; and although advances in the ability of countries to produce scientific information on aging are recognized, this capacity is still limited and weak to generate evidence for decision making. In 2019, 22 countries reported to PAHO to have some information on this issue, and 15 countries reported some research on health and well-being of the elderly. It is outstanding to note that several countries have supported and developed longitudinal-type population research, with a high level of comparability among them, which may mean that in the near future, the national and regional capacity to generate evidence for decision making will be substantially improved.

The World Health and Aging Report highlighted that “*although it is required that monitoring efforts and population research take older people more into account, specific population research on these people is also required, in order to determine the levels and the distribution of functional capacity and intrinsic capacity, how these change over time, and what the needs are for health care and support, and to what extent they are met.*” SABE Colombia, whose results are published in this issue, is a clear example of these investigations ^1^.

The World Health Organization and its Member States have begun the process of developing the proposal of the 2020-2030 Decade as a Decade of Aging, which should be approved in 2020. Two of its ten priorities: “the collection of better global data about Healthy Aging” and “promoting research that addresses the current and future needs of older people” come true in this issue of ***Colombia Médica*** .

Thanks to all who have made this a reality.
