# Supplementary material for: Research: a priority for the decade of healthy aging
Source: Colomb Med (Cali). 2019 Jun 30;50(2):50–1. doi: 10.25100/cm.v50i2.4613 (PMC6774578; doi:10.25100/cm.v50i2.4613)
Supplement: Translation

# Translation: La investigación una prioridad para la década del envejecimiento saludable

Es una gran oportunidad ser parte de la publicación de este número especial dedicado al envejecimiento y a la salud, agradezco a los editores por esta oportunidad. La relevancia de esta publicación no es casual, el envejecimiento poblacional es sin duda uno de los retos más formidables que la salud pública deberá enfrentar en este siglo. Es un reto global, pero para las Américas, y especialmente para América Latina y el Caribe, es ya de una actualidad ineludible. 

La última década ha sido importante para la salud pública y el envejecimiento en las Américas. Con orgullo podemos afirmar que ninguna Región avanzó tanto como la nuestra, en estos diez años. En el 2009, los Ministros de Salud de las Américas, fueron pioneros en aprobar un plan de acción en Envejecimiento y Salud, el primero en el mundo, también las Américas fue la primera; y hasta ahora, la única región del mundo en aprobar un mecanismo de protección para los derechos de las personas mayores, la Convención Interamericana de los Derechos de las Personas Mayores en el 2015. 

Muchos países en la Región han reportado resultados y avances importantes en el trabajo sobre la salud de las personas mayores y han tenido un papel relevante en la aprobación del Plan Global de Envejecimiento y Salud en el 2016 en la Asamblea Mundial de la Salud, y hoy toman el liderazgo en la promoción de la Declaración de la Década del Envejecimiento Saludable. Sin embargo, la profundidad y rapidez de la transición demográfica en las Américas ha desbordado hasta hoy nuestros esfuerzos. Si en el 2017 la población en nuestro continente tenía un 14,6% de personas con 60 años y más, para el 2050 esta cifra alcanzara un 25%. Se espera que la esperanza de vida de la Región siga incrementándose para todas las edades. La esperanza de vida al nacer hoy es de 77.07 años, la esperanza de “vida geriátrica”, cuando se arriba a los 60 años, es de vivir 22.38 años más, y una persona que llega a los 80 años hoy en las Américas vivirá como promedio 9.41 años más. Pero, quizás lo más importante es que esta evolución le tomará América Latina y el Caribe 35 años; casi la mitad del tiempo que le tomó en otras regiones: a Europa le tomó 65 años y a Estados Unidos y Canadá, 75 años ^1^.

Aunque reducir la mortalidad y el prolongar la vida debe ser reconocido como uno de los grandes resultados del desarrollo social y de la salud pública, no todo han sido buenas noticias. En el 2015, se estimó que la “**esperanza de vida saludable”** en la Región era de 66.45 años, con una diferencia de más de 10 años con respecto a la esperanza de vida (77.07 años). En las Américas, los años vividos con discapacidad aumentó 12.6 % desde el 2009. Aunque hay diferencias notables entre países, en todos ha aumentado la brecha entre ambos indicadores. Varios estudios avalan que más de ocho millones de personas de 60 años o más son dependientes, lo cual representa más del 1% de la población total de la región, y un 12% de los individuos con más de 60 años. Se estima que para el 2050 esta cifra se triplicará y estará entre 27 y 30 millones ^2^. 

A pesar de los importantes avances en la Región, en la última década, la rapidez de la transición demográfica, con otras muchas prioridades que atender y en un contexto socioeconómico complejo, exigirá una adecuación mucho más rápida de los sistemas de salud. La llamada “ventana de oportunidad demográfica” se estrecha rápidamente para las Américas; y aunque el sentido de prioridad sobre el tema ha crecido, deberá hacerse un esfuerzo mayor para atender esta transición. Lo anterior necesitará de acciones e intervenciones de la OPS/OMS, los Estados Miembros, la Secretaría y otros socios estratégicos que permitan que la longevidad y el envejecimiento sean un resultado positivo del desarrollo sostenible de las Américas ^3^. Lo anterior implica que deberán redoblarse los esfuerzos para lograr disminuir esta brecha, se debe aprender sobre las nuevas necesidades para impactar las condiciones que provocan grandes periodos de tiempo viviendo con discapacidad y dependencia. 

En mis ya más de 30 años de carrera asociados a este tema, he sido testigo del esfuerzo en América Latina y el Caribe por crear una base científica cada vez más sólida que contribuirá a la toma de decisiones informadas y basadas en evidencias por nuestros gestores en salud y para evaluar que estas intervenciones realmente generan mejoras importantes en la salud y el bienestar de la población envejecida. Pero ha faltado reconocer con mayor fortaleza, la relevancia e influencia de estos esfuerzos, como sustento de nuestros avances en la política pública sobre envejecimiento. Esta publicación es un gran espacio para reconocer los esfuerzos pioneros con el estudio SABE (Salud, Bienestar y Envejecimiento) bajo la conducción y liderazgo de la Dra. Martha Peláez que realizó la OPS y otros socios estratégicos a principios de este siglo ^4^. Este estudio multinacional fue durante muchos años una de las fuentes más importantes de la base científica, que con datos y evidencias sostuvo la necesidad del avance del trabajo en la salud de las personas mayores en todos los niveles y sectores de la sociedad. Su influencia se extendió y estimuló la realización de estudios nacionales de trascendental importancia, de los cuales los resultados de esta publicación en la revista Colombia Medica, es un ejemplo valioso. 

El trabajo es todavía perfectible y aunque se reconocen avances en la capacidad de los países en producir información científica en envejecimiento, esta capacidad todavía es limitada y débil para generar evidencia para la toma de decisiones. En el 2019, 22 países reportaron a la OPS tener alguna información en este tema y 15 países reportaron alguna investigación sobre salud y bienestar de las personas mayores. Es excelente remarcar que varios países han apoyado y desarrollado investigaciones poblacionales de tipo longitudinal, con alto nivel de comparabilidad entre ellas, que puede significar que en el futuro cercano la capacidad nacional y regional de generar evidencias para la toma de decisiones será sustancialmente mejorada.

El Informe Mundial de la Salud y el Envejecimiento, resaltó que *“aunque es preciso que los esfuerzos de seguimiento y las investigaciones poblacionales tengan más en cuenta a las personas mayores, también se requieren investigaciones poblacionales específicas sobre estas personas para determinar cuáles son los niveles y la distribución de la capacidad funcional y la capacidad intrínseca, cómo estas cambian con el tiempo y cuáles son las necesidades de asistencia sanitaria, cuidados y apoyo, y en qué medida se satisfacen” .* SABE Colombia, cuyos resultados se publican en este número es un claro ejemplo de estas investigaciones ^1^. 

La Organización Mundial de la Salud y sus Estados Miembros han comenzado el proceso de desarrollo de la propuesta de la Década 2020-2030 como Década del Envejecimiento que deberá ser aprobada en el 2020. Dos de sus diez prioridades: “la *recopilación de mejores datos globales sobre el Envejecimiento saludable*” y la de “*promover investigaciones que aborden las necesidades actuales y futuras de las personas mayores”* se hacen realidad en este número de Colombia Médica. 

Gracias a todos los que lo han hecho esto una realidad.
